# A Comprehensive Review of Robinetin: Distribution, Biological Activity and Pharmacokinetic Parameters

**DOI:** 10.3390/ijms26199546

**Published:** 2025-09-30

**Authors:** Katarzyna Jakimiuk

**Affiliations:** Department of Biology and Pharmacognosy, Faculty of Pharmacy with the Division of Laboratory Medicine, Medical University of Białystok, Mickiewicza 2a, 15-230 Białystok, Poland; katarzyna.jakimiuk@umb.edu.pl; Tel.: +48-85-748-51-30

**Keywords:** robinetin, polyhydroxyflavone, distribution, biological activity, pharmacokinetic

## Abstract

Robinetin, a naturally occurring polyhydroxylated flavonol, has gained attention due to its broad spectrum of biological activities and potential therapeutic applications. This review presents a comprehensive summary of the current knowledge concerning the natural occurrence, extraction, spectroscopic characterization, and pharmacological properties of robinetin. Ethnobotanical evidence highlights its presence in various medicinal plants, particularly within the Fabaceae family, where it contributes to traditional treatments of infections, inflammation, and metabolic disorders. Robinetin exhibits diverse bioactivities, including antiviral, antibacterial, antiparasitic, antioxidant, anti-mutagenic, and enzyme-inhibitory effects. Notably, it inhibits HIV-1 integrase and acetylcholinesterase and demonstrates moderate antiproliferative activity in cancer cell lines. Despite limited water solubility, its redox behavior and metal-chelating capabilities support its antioxidant potential. Recent in vivo studies indicate its hepatoprotective and metabolic regulatory effects. Additionally, computational models reveal promising interactions with molecular targets such as CDK1. Collectively, these findings underscore the multifaceted therapeutic potential of robinetin and advocate for further pharmacokinetic and clinical investigations to validate its efficacy as a lead compound for the development of phytochemically derived pharmaceuticals.

## 1. Introduction

**Figure 1:**
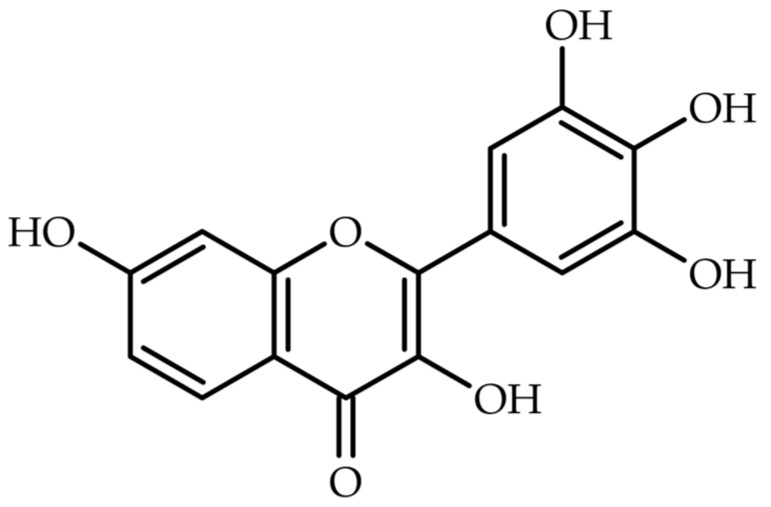
Chemical structure of robinetin.

Plants are an infinite source of bioactive substances where flavonoids remain one of the most important chemicals with health-beneficial potential [1]. Flavonoids are divided into groups according to their substitution pattern and primarily are composed of phenols in their free form or glycosides [2]. Polyhydroxylated flavonoids (PHF) are a class of polyphenolic compounds characterized by multiple hydroxyl groups, which confer diverse and significant biological activities [3,4]. The biological activities of polyhydroxylated flavonoids are significantly influenced by their structural features. The number and position of hydroxyl groups, degree of glycosylation, and presence of other substituents affect their solubility, bioavailability, and interaction with biological targets [4]. Also, the diversity in physicochemical properties of this class of compounds influence their biological activity, solubility, and bioavailability which is essential to understanding their mechanisms of action and optimizing their therapeutic applications [5]. Polyhydroxylated flavonoids generally exhibit poor water solubility due to their hydrophobic aromatic ring; however, aglycone forms are more lipophilic, facilitating their penetration through lipid bilayers and membranes [6]. Furthermore, these flavonoid derivatives are effective metal chelators, particularly for transition metals (e.g., iron, copper). Chelation occurs via hydroxyl groups, especially in positions C3, C4, and C5 on the flavonoid backbone. This property is crucial for their antioxidant effects, as it prevents metal-catalyzed oxidative reactions [7,8]. Another important chemical reactivity of flavonoids with unsubstituted hydroxyl groups is redox properties. The hydroxyl groups in flavonoids undergo redox cycling, allowing them to donate electrons to neutralize free radicals. This redox capability is enhanced in flavonoids with catechol structures in their B-ring [9,10]. One of polyhydroxy flavones occurring in the plant kingdom is robinetin (norkanugin, 5-hydroxyfisetin, 5-deoxymyricetin) which belong to the flavonol subclass (Figure 1). Robinetin consists of a flavonoid skeleton with hydroxyl groups at the 3, 5, 7, 3′, and 4′ positions [11]. Available studies suggest that robinetin exhibit diverse biological activities and may be promising antiviral, anti-inflammatory, antioxidant or anticancer therapeutic agent [12,13,14].

Although various biological activities of robinetin have been established, no clearly organized review articles are available. Thus, this paper gives an outline of the findings on the techniques used for the analysis, isolation, and separation of robinetin, as well as described biological properties and therapeutic activities of this compound.

## 2. Methodology

A systematic literature search was conducted using a predefined query across key bibliographic platforms. The following resources were used: Scopus; (https://www.elsevier.com/ (accessed on 9 September 2025)); Google Scholar (https://scholar.google.com/ (accessed on 9 September 2025)); PubMed/MEDLINE (https://pubmed.ncbi.nlm.nih.gov/ (accessed on 9 September 2025)); Web of Science (SCI-EXPANDED) (https://www.webofscience.com/ (accessed on 9 September 2025)); Taylor & Francis Online (https://taylorandfrancis.com/online/taylor-francis-online/ (accessed on 9 September 2025)); Wiley Online Library (https://onlinelibrary.wiley.com/ (accessed on 9 September 2025)); EBSCO Discovery Service (EDS) (https://search.ebscohost.com/ (accessed on 9 September 2025)); REAXYS (https://www.reaxys.com/#/search/quick/query (accessed on 9 September 2025)); and ScienceDirect (Elsevier) (https://www.sciencedirect.com/ (accessed on 9 September 2025)). The chemical structure validation was achieved via cross-referencing entries in PubChem and REAXYS. TITLE-ABS-KEY fields in the specified databases were queried using designated keywords, either individually or in various logical combinations, in accordance with each database’s search constraints: “robinetin”, “robinetin derivatives”, “polihydroxyflavonoids”, “biological activity”, “natural compound”, “isolation and identification”, “chemical characterization”, “chemical analysis”, “metabolism”, “chemical and physical properties”. Inclusion criteria encompassed English-language studies published between 1933, marking the first documented mention of robinetin, and 2025 (September). The search process featured a two-stage screening—first assessing titles and abstracts, then evaluating full-text articles. Further relevant studies were located through review article bibliographies and cross-referencing of cited works. The chemical structure of robinetin was drawn using ACD/ChemSketch (2014.1.5 Freeware Version).

## 3. Natural Occurrence of Robinetin

Table 1 presents a compilation of ethnopharmacological data concerning robinetin-containing plant species, with a predominant representation from the Fabaceae family, alongside members of the Boraginaceae, Asteraceae, Loranthaceae, Nelumbonaceae, Brassicaceae, Araceae and Moringaceae families. The documented species exhibit a wide range of traditional medicinal applications, often associated with specific plant parts such as bark, leaves, seeds, and roots. For instance, *Acacia mearnsii* is traditionally utilized for the treatment of microbial infections, whereas *Albizia lebbeck* demonstrates a broad ethnomedical profile, with its leaves employed in managing ulcers, night blindness, respiratory and dermatological conditions, as well as envenomations, leprosy, gonorrhea, and pharyngeal disorders [15,16]. In *Adenanthera pavonina*, both seeds and leaves, is known for its application in treating boils and inflammatory states [17]. *Cordia myxa*, a representative of the Boraginaceae family, has traditionally been applied in the treatment of wounds, boils, tumors, gout, and ulcers, despite the unspecified plant part used [18]. *Cosmos caudatus*, belonging to the Asteraceae family, exhibits multifaceted therapeutic uses, with its leaves employed for antidiabetic, antihypertensive, anti-inflammatory, hepatoprotective, and antimicrobial purposes, as well as for enhancing blood circulation, strengthening bones, cooling the body, and decelerating aging processes [19]. The root of *Entada africana* is traditionally recognized for its role in treating inflammatory diseases, hepatitis, bronchitis, and cough, in addition to its utility in wound healing, diuresis, and as an anti-gonococcal and anti-syphilitic agent [20]. The bark of *Intsia bijuga* is associated with the management of rheumatism, dysentery, urinary tract infections, asthma, diabetes, ulcers, and skeletal fractures [21]. *Robinia pseudoacacia* bark is traditionally utilized for its laxative, antispasmodic, and diuretic properties [22]. Collectively, the data underscore the ethnomedical significance of these species and highlight their diverse pharmacological potential within traditional healing systems.

Thus, Table 1 synthesizes current knowledge regarding the botanical sources, tissue-specific localization, and geographic distribution of robinetin, highlighting its role as both a taxonomic marker and bioactive metabolite in various plant species worldwide.

## 4. Techniques for the Analysis of Robinetin in Plant Material

The extraction of robinetin from *Robinia pseudoacacia* wood was investigated using three conventional methods: Soxhlet extraction, ultrasonic extraction, and maceration with stirring. The solvents used for the extraction were acetone, methanol and ethanol, each containing 10% of water (*v*/*v*). Among these, Soxhlet extraction proved to be the most efficient, yielding the highest concentrations of robinetin as well as the related flavanonol dihydrorobinetin. In contrast, ultrasonic extraction and maceration provided lower yields overall, although ultrasonic extraction achieved relatively high recovery in a shorter time, making it advantageous in terms of reduced solvent and energy consumption. With respect to solvents, the amounts of robinetin extracted did not differ substantially between acetone, ethanol, and methanol. Overall, Soxhlet extraction with aqueous acetone was identified as the optimal laboratory-scale method for obtaining robinetin [63].

Chromatography currently represents a fundamental technique in the field of separation and analytical sciences, and its widespread adoption across research institutions and the pharmaceutical industry underscores its central role in modern analytical methodologies [64]. Column chromatography was employed to separate robinetin from the leaves of *Robinia pseudoacacia*. The extraction was carried out using ethanol, followed by purification on Sephadex LH-20. Further separation was performed with reversed-phase (RP) silica gel columns using a methanol–water system (9:1). Finally, a Lobar column with the same solvent system (MeOH:H_2_O, 9:1) was applied to achieve effective isolation of robinetin [62].

High-performance liquid chromatography (HPLC), unlike TLC, offers several critical advantages in analytical applications, including the ability to perform both qualitative and quantitative determinations with high efficiency, sensitivity, and rapid separation [65]. As a continuously evolving technique, HPLC has demonstrated broad applicability and plays a key role in the analysis of plant-derived extracts and fractions. Its utility in the detection and isolation of robinetin is well-established, with specific analytical conditions outlined in Table 2.

Sanz and co-workers using spectroscopic and spectrometric data of peaks in HPLC chromatograms seasoned and toasted *Robinia pseudoacacia* heartwood extracts identified robinetin with λ_max_ 254 and 364 nm, MS/MS *m*/*z* [M-H]—301, 273, 245, 229, 135, 91 [57].

## 5. Biological Activities of Robinetin

Obtaining bioactive compounds from medical plants is fundamental to the production of drugs of the plant origin. Thus, Table 3 summarizes biological activities of robinetin in vitro, in vivo and in silico studies. Considering performed spectroscopic analyses, complemented by molecular modeling calculations, provide important insights into robinetin binding within a human serum albumin (HSA) matrix, highlighting its potential utility as a sensitive multiwavelength fluorescent probe. Such studies are expected to facilitate the rational screening and design of structurally optimized flavonoid-based compounds, representing a promising class of phytochemicals with significant potential as alternatives to conventional therapies [66,68]. Another study also showed that robinetin inhibits lipid peroxidation in a dose-dependent manner and prevents non-enzymatic glycosylation of hemoglobin [69].

### 5.1. Antiviral Activity

To study the antiviral activity, Fesen and co-workers investigated the inhibition of HIV-1 integrase by robinetin. It was revealed that robinetin inhibited 3′—processing and strand transfer with IC_50_ = 5.9 ± 1.9 µM and 1.6 ± 0.7 µM [70].

Moreover, robinetin has been reported to exhibit antiviral potential against SARS-CoV-2 based on in silico molecular docking studies, demonstrating a binding affinity of −8.3 kcal/mol for the main protease (Mpro) and −7.6 kcal/mol for the spike glycoprotein. These interactions suggest potential inhibition of viral replication and interference with host cell entry mechanisms. The docking results indicate that robinetin forms up to five hydrogen bonds and multiple hydrophobic contacts within the active sites of these proteins, stabilizing the ligand–protein complex [73].

### 5.2. Antibacterial Activity

Robinetin was also studied as an antibacterial agent against human skin bacterium bacteria *Proteus vulgaris* and *Staphylococcus aureus*, where MIC after its exposure gain 100 µg/mL in both bacterium [75].

### 5.3. Antiparasitic Activity

To study the antiparasitic activity of robinetin, Tasdemir et al. performed in vitro assay using *Leishmania donovani* strain MHOM/ET/67/L82, *Trypanosoma brucei rhodesiense* SSTIB 900, and *Trypanosoma cruzi* C2C4 with IC_50_ values of 5.9 µg/mL, 5.3 µg/mL and above 30 µg/mL, respectively [77].

### 5.4. Antioxidant Activity

Using the density functional theory (DFT) with B3LYP functional and 6–311++G (d, p) basis set, the antiradical activity of robinetin has been investigated. Calculations were carried out both in the gas phase and with consideration of solvent effects: water, dimethyl sulfoxide (DMSO), methanol, and benzene. Three mechanisms were examined—hydrogen atom transfer (HAT), single-electron transfer followed by proton transfer (SET-PT), and sequential proton loss electron transfer (SPLET)—to evaluate radical scavenging ability and identify the most likely antioxidant action pathway. Final findings indicated that the 4′-OH group is the most reactive site. Among the evaluated pathways, the HAT mechanism proved to be the most energetically favorable [78,93]. According to Huguet and co-workers, robinetin possesses superoxide scavenging activity comparable to that of previously known scavengers of superoxide anions, such as morin, rutin or luteolin [94].

### 5.5. Anticancer Activity

Numerous studies have explored the potential of robinetin as an anticancer agent. Unfortunately, this polyhydroxylated flavonoid exhibits limited activity against two human melanoma cell lines, C32 and A375, as the IC_50_ values in both MTT and NRU assays after 24, 48, and 72 h of incubation exceeded the highest tested concentration (200 µM) [81]. The effect of robinetin (6.25–200 µM) was also examined in an oral squamous carcinoma (SCC-25) cell line. In both, MTT and NRU tests IC_50_ was above 200 µM [80].

Robinetin was evaluated for their inhibitory efficacy against CDK1 (cyclin-dependent kinase 1) through molecular docking studies. CDK1, a key regulator of the cell cycle component central to the uncontrolled proliferation of malignant cells, has been reportedly implicated in colorectal cancer. The authors predicted that robinetin may be active in terms of carcinogenicity and mutagenicity [82].

Chang et al. applied robinetin topically at a dose of 2.5 µmol. It exhibited no intrinsic tumor-initiating activity on mouse skin. When administered 5 min prior to 200 nmol of B[a]P 7,8-diol-9,10-epoxide-2 (B[a]P), it resulted in only a 16–24% reduction in tumor incidence, which was not statistically significant. Similarly, robinetin showed little or no effect on the tumor-initiating activity of 50 nmol of B[a]P. In contrast, systemic administration of 1.4 µmol intraperitoneally to preweaning mice did not induce tumors over a 9–11 month observation period, but pretreatment before exposure to 30 nmol of diol-epoxide produced a substantial 44–75% inhibition of pulmonary tumor formation, indicating partial chemopreventive potential [83].

In another study, robinetin was tested for its effect on melanogenesis in HMV II human melanoma cells. The compound showed no melanogenesis-promoting activity under the experimental conditions [95].

Robinetin was tested for its ability to modulate multidrug resistance in Colo 320 human colon cancer cells expressing MDR1/LRP. The compound exhibited only marginal effects on Rhodamine 123 accumulation, indicating limited potential in reversing MDR (multi-drug resistance). Moreover, robinetin acted as a weak apoptosis inducer in both drug-resistant and drug-sensitive colon cancer cells, without significant differences between the two cell lines [84]. Also, in SW480 and T84 colon carcinoma cells robinetin possesses weak anticancer activity (IC_50_ = 100 µM) [85].

### 5.6. Anti-Mutagenic Activity

Anti-mutagenic activity was evaluated using the *Salmonella typhimurium* mutagenicity assay by assessing the ability of the test compounds to inhibit mutation rates induced by known chemical mutagens, including methyl-nitrosourea (MNU), methyl-n-nitro-N-nitrosoguanidine (MNNG), benzo-*γ*-pyrene (BaP), and 2-aminoanthracene (2-AA). Robinetin caused 11%, 6%, 1.2% and 87% inhibition of mutagenicity induced by MNU, MNNG, BaP and 2-AA, respectively [86].

Since aflatoxin B_1_ is highly toxic and mutagenic substance, Bhattacharya and Firozi evaluated the effect of robinetin and other flavonoids on microsome catalyzed reactions of aflatoxin B_1_, leading to activation and DNA adduct formation. According to their results, robinetin is one of the most active compounds in inhibiting microsome-mediated activation of aflatoxin B_1_ (11.4% of control) and subsequent DNA adduct formation (7.7% of control). Its strong activity suggests that robinetin may counteract the carcinogenic effects of aflatoxin B_1_ through modulation of microsomal enzyme function [87].

Robinetin was tested for mutagenicity in the *Salmonella*/*mammalian* microsome assay using multiple tester strains. It exhibited low frameshift mutagenic activity, with a revertant value of 0.06 revertants/nmol in strain TA98, indicating weak mutagenic potential compared to quercetin and kaempferol. Like other flavonols, robinetin showed significant activation by Aroclor 1254-induced rat-liver microsome preparations. These findings suggest that the mutagenic activity of robinetin is modest and dependent on microsomal enzyme-mediated activation [96]. In another study, robinetin effectively reduced mutagenicity induced by tert-butyl hydroperoxide (BHP) and cumene hydroperoxide (CHP) in *Salmonella typhimurium* TA102. Its antimutagenic activity was comparable to that of other polyhydroxylated flavonols such as quercetin, fisetin, and myricetin, with ID_50_ values ranging from 0.25 to 1.05 μmol per plate. Structural modifications, such as hydrogenation, led to a marked loss of antimutagenic potential, highlighting the importance of the double bond between carbons 2 and 3. These findings indicate that the protective effects of robinetin are largely attributable to its radical scavenging properties [97].

### 5.7. Anti-Necroptosis Activity

The anti-necroptotic potential of flavonoid inhibitors was assessed by measuring cell viability following TSZ-induced necroptosis. Robinetin demonstrated a significant cytoprotective effect, markedly enhancing cell viability at a concentration of 50 μM. In HT-29 cells, robinetin restored survival to levels comparable to those observed in untreated controls. Notably, robinetin maintained its anti-necroptotic efficacy across a range of TSZ-induced necroptotic conditions in both mouse embryonic fibroblasts (MEFs) and HT-29 cells, with observed survival rates ranging from 18% to 56%, corresponding to baseline cell viability. These findings indicate that robinetin confers potent protective effects against necroptotic cell death in vitro. Additionally, ADP-Glo assay revealed RIPK1 inhibition by robinetin (IC_50_ = 43.8 μM) [88].

### 5.8. Enzyme Inhibition Activity

Acetylcholinesterase inhibitory activity can be a useful tool in the treatment of Alzheimer’s disease (AD). Robinetin was evaluated for its inhibitory activity using an in vitro assay, revealing a half-maximal inhibitory concentration (IC_50_) of 456.48 ± 2.57 µM [89]. In another study Hanaki and co-workers identify bioactive natural products in crude drugs that inhibit A*β*42 aggregation and that could be applied to future AD therapies. They selected 6 flavonoids, including robinetin, which suppressed A*β*42 aggregation [44].

Robinetin demonstrated strong inhibitory activity against both MRP1 and MRP2 in MDCKII transfected cells. The compound exhibited IC_50_ values of 13.6 µM for MRP1 and 15.0 µM for MRP2, indicating comparable potency toward both transporters. Detailed kinetic analysis revealed that robinetin acts as a competitive inhibitor with respect to calcein, with apparent inhibition constants (K_i_) of 5.0 µM for MRP1 and 8.5 µM for MRP2. These findings highlight robinetin as a potent and structurally significant flavonoid in modulating multidrug resistance protein activity [91]. Robinetin also possesses inhibitory potency against NADH-oxidase with IC_50_ value 19 nmol/mg protein [98].

### 5.9. Activity in the Liver Diseases

To evaluate the ameliorating effect of robinetin on the significant pathogenic features of metabolic failure in the liver and to identify the underlying molecular mechanism, Song et al. used AML-12 hepatocytes untreated and treated with OPA mixture (800 μM oleic acid and 150 μM palmitic acid) and five-week-old male C57/BL6 mice (divided into 4 groups: control, Western diet-feed group (WD), WD with 0.025% robinetin group, and WD with 0.05% robinetin. The researchers proved that robinetin caused suppression of TG accumulation in AML-12 cells, by abrogating the mRNA expression of lipogenesisrelated genes. Also, the liver tissues in the robinetin-supplemented mice exhibited reduced lipid droplet accumulation compared with that of the WD-fed (Western diet) mice. The OGTT results revealed that the blood glucose levels in robinetin-supplemented groups were lower after oral glucose loading. On the other hand, in the mice model it was established that the liver size in mice fed the robinetin-supplemented diet was similar to that in mice in the control group. Its strong activity is associated with the presence of adjacent tri-hydroxyl groups, which promote a substantial rate of auto-oxidation. During cyanide-stimulated oxidation, robinetin contributed to the non-enzymatic generation of superoxide anions, a process attenuated by superoxide dismutase [91].

Summarizing, the information about biological activity of robinetin, the available data are derived from highly heterogeneous experimental models, which complicates direct comparison of reported activities. Many studies rely on biochemical assays that yield low-micromolar IC_50_ values, whereas cell-based evaluations often indicate only weak effects at much higher concentrations (>200 µM), likely reflecting differences in compound solubility, stability, membrane permeability, and protein binding. Computational approaches (docking, DFT) offer mechanistic insights but require validation with orthogonal biochemical and cellular assays. Finally, very few studies have incorporated pharmacokinetic or metabolism data; yet microsome-dependent activation results suggest that metabolites may substantially alter biological outcomes. Together, these factors underscore the need for standardized protocols, careful control of redox conditions, and integrated biochemical, cellular, and in vivo studies to more reliably define the pharmacological potential of robinetin.

## 6. ADMET of Robinetin

Analysis of urine samples from rats administered robinetin (200 mg/rat) as well as ether extracts from incubation mixtures (10 mg/tube), demonstrated the presence of substantial amounts of unmetabolized robinetin. It is worth mentioning that in this study the metabolism of tricine and morin was also examined; in the sample with robinetin, no metabolites formed after the metabolism of this flavonoid were found. The results indicate that robinetin is resistant to bacterial catabolism [99].

Additionally, through Deep-PK Predictions analysis, the ADMET (absorption, distribution, metabolism, excretion and toxicity) properties of robinetin can be computationally estimated [100]. The selected pharmacokinetic parameters and toxicity properties of robinetin (C1=CC2=C(C=C1O)OC(=C(C2=O)O)C3=CC(=C(C(=C3)O)O)O) are given in Table 4.

The clinical use of robinetin may be significantly limited by its poor oral bioavailability and short half-life. Predictive data indicate that its oral bioavailability in humans is low, suggesting limited systemic exposure following oral administration. This reduced absorption, combined with a predicted half-life of less than 3 h, means that therapeutic plasma concentrations may not be maintained over long periods, necessitating frequent dosing. Such pharmacokinetic limitations may hinder patient compliance and reduce the overall therapeutic efficacy of the compound. To address these challenges, several strategies could be considered. Formulation approaches, such as the use of nanoemulsions, liposomes, or solid lipid nanoparticles, may enhance solubility, protect the compound from premature metabolism, and improve gastrointestinal absorption. Co-administration with permeability enhancers or inhibitors of efflux transporters could also be explored to increase oral exposure. Alternatively, chemical derivatization, including the development of prodrugs or structural analogs with improved pharmacokinetic properties, could extend systemic half-life and bioavailability. Furthermore, long-acting delivery systems (e.g., polymer-based depots or sustained-release formulations) may help overcome the rapid clearance. Overall, rational optimization through formulation science and medicinal chemistry will be essential to unlock the therapeutic potential of robinetin [101].

## 7. Conclusions

Robinetin, a polyhydroxylated flavonol, emerges as a bioactive compound of significant pharmacological relevance, supported by evidence from in vitro, in vivo, and in silico studies. Its wide spectrum of activities, including antiviral, antibacterial, antiparasitic, antioxidant, anti-mutagenic, anti-necroptotic, and enzyme-inhibitory effects, highlights its multifaceted therapeutic potential. Despite its relatively modest anticancer efficacy in certain cell models, robinetin demonstrates promising activity in modulating multidrug resistance proteins and protecting against liver metabolic failure, suggesting value in chemopreventive and metabolic disease contexts. Structural features, particularly hydroxyl group substitutions, play a decisive role in its redox activity, metal chelation, and radical scavenging capacity, underscoring the importance of structure–activity relationships. However, limitations such as poor water solubility and limited pharmacokinetic data constrain its direct application. Thus, future research on robinetin should prioritize comprehensive in vivo pharmacokinetic studies to validate current computational predictions and clarify its absorption, metabolism, and systemic distribution. Structural modifications or formulation strategies, such as prodrug design or nanocarrier-based delivery, are needed to improve its poor aqueous solubility and oral bioavailability. Additionally, investigations into synergistic interactions with other flavonoids could enhance its antioxidant, antiviral, or anticancer efficacy. Finally, preclinical and translational studies are essential to elucidate molecular mechanisms and assess the therapeutic potential of robinetin in clinical settings.

## Figures and Tables

**Table 1:** Plants containing robinetin and its traditional use.

Plant	Family	Plant Part	Traditional Use	References
*Acacia* *mearnsii*	Fabaceae	bark	microbial infections	[15,23,24]
*Albizia* *lebbeck*		leaves	treatment of ulcers, night blindness, respiratory disorders, skin disorders, snake, bite, piles, leprosy gonorrhea, scorpion bite, cough, pharyngitis	[16,25]
*Adenanthera* *pavonina*		seeds, leaves	treatment of boils and inflammations	[17,26]
*Burkea* *africana*		bark	not found	[27]
*Cordia* *myxa*	Boraginaceae	not given	treatment of wound, boils, tumors, gout and ulcer; blood purifier and febrifuge	[18,28]
*Cosmos* *caudatus*	Asteraceae	leaves	anti-diabetic, anti-hypertensive, anti-inflammatory, hepatoprotective, antimicrobial, blood circulation booster, bone strengthener, body-cooling agent and anti-aging agent	[19,29]
*Dendrophthoe* *pentandra*	Loranthaceae	leaves	immunological disorders and cancers	[30]
*Entada* *africana*	Fabaceae	root	treatment of inflammatory diseases, hepatitis, bronchitis and cough and wound-healing, diuretic, anti-gonococci and anti-syphilitic agent	[20]
*Erucaria* *microcarpa*	Brassicaceae	aerial parts	not found	[31]
*Gliricidia* *sepium*	Fabaceae	bark	anti-microbial, antibacterial, anti-inflammatory, thrombolytic, antisickling, wound healing agent	[32,33]
*Gliricidia* *maculata*			herbal galactogogue	[32,34]
*Lemna* *minor*	Araceae	leaves	human food	[35,36]
*Intsia* *bijuga*	Fabaceae	bark	rheumatism, dysentery, urinary tract infections, asthma, diabetes, ulcers, fractures	[21,37,38,39,40]
*Millettia stuhlmannii*			not found	[41]
*Moringa* *oleifera*	Moringaceae	leaves	paralysis, helminthiasis, sores and skin infections	[42,43]
*Nelumbo* *nucifera*	Nelumbonaceae	leaves	hyperlipidemia, hematemesis, metrorrhagia, fever treatment, release skin inflammatory symptoms	[44,45]
		seeds	tissue inflammation, cancer, diuretics, skin diseases and as poison antidote	[44,46]
		root	circulatory system disorders, diarrhea, insomnia, fever, body heat imbalance and gastritis	[44,45]
*Robinia* *pseudoacacia*	Fabaceae	bark	laxative, antispasmodic, diuretic agent	[22,47,48,49,50,51,52,53,54,55,56,57,58,59,60]
		leaves		[61,62]

**Table 2:** High-performance liquid chromatography in the identification of robinetin.

Extract	Column	Mobile Phase	Conditions	Ref.
***Robinia pseudoacacia*** **bark**				
50% MeOH	Hypersil ODS C18	0.1% PhA (A) MeOH; PhA 0.1% (B)	100–95% A: 0–50 min 95–70% A: 50–85 min 70–0% A: 85–105 min	[66]
Et_2_O, EtOAc lyophilizates (in 50% MeOH)				[57]
Et_2_O, EtOAc			100–85% A: 0–20 min 85–75% A: 20–30 min 75–50% A: 30–50 min 50–0% A: 50–70 min	
90% acetone	Thermo Accucore C18	H_2_O + 0.1% FA (A); MeOH + 0.1% FA (B)	5–95% of solvent B	[67]
***Lemna minor*** **leaves**				
50% MeOH	Poroshell 120 EC-C18; ZIC-HILIC	10 mM NH_4_OAC in 9:1 (*v*/*v*) H_2_O–ACN (A); 10 mM NH_4_OAC in 1:9 (*v*/*v*) H_2_O–ACN (B) (RPLC); ACN (C); H_2_O (D) (HILIC)	Binary pump 1: 100% A: 0–7 min 100–50% A: 7–13 min 50–0% A: 13–33 min 100% A: 33–58 min Binary pump 2: 100% C: 0–7 min 100–60% A: 7–13 min 100% C: 13–53 min	[35]
***Intsia bijuga*** **bark**				
EtOH	Accucore C18 column	H_2_O + 0.1% FA (A); ACN + 0.1% FA (B)	5–25% B: 0–3 min 25–55%: 3–22.5 min 55–95%: 22.5–25 min 95% B: 25–28 min 5% B: 29–30 min	[40]

MeOH—methanol; Et_2_O—diethyl ether; EtOAc—ethyl acetate; PhA—phosphoric acid, ACN—acetonitrile; NH_4_OAC—ammonium acetate; FA—formic acid.

**Table 3:** Biological activities of robinetin in vitro, in vivo and in silico models.

Experimental Model	Exposure/ Incubation	Concentration	Efficacy	Ref.
**ANTIVIRAL**				
HIV integrase catalytic assays	1 h incubation	4 µL of sample	Cleavage IC_50_ = 5.9 ± 1.9 µM Integration IC_50_ = 1.6 ± 0.7 µM	[70,71,72]
SARS-CoV-2 virtual screening	-	-	MM-GBSA ΔGBind = −47.544 kcal/mol Binding affinity (Mpro) = −8.3 kcal/mol Binding affinity (spike glycoprotein) = −7.6 kcal/mol	[73]
SARS-CoV-2 M^pro^ inhibition (FRET)	-	10 µM	% of inhibition at 10 µM: 5.96 IC_50_ > 10	[74]
**ANTIBACTERIAL**				
*Proteus vulgaris,* *Staphylococcus aureus*	1 h incubation	not given	MIC (µg/mL): *P. vulgaris*: 100 *S. aureus*: 100	[71,75]
**ANTIPARASITIC**				
*Leishmania* *donovani* MHOM/ET/67/L82	72 h incubation	30 to 0.041 µg/mL	IC_50_ = 5.9 µg/mL	[76,77]
*Trypanosoma brucei rhodesiense* SSTIB 900	72 h incubation	90 to 0.123 µg/ml	IC_50_ = 5.3 µg/mL	[77]
*Trypanosoma cruzi* C2C4	96 h incubation	not given	IC_50_ > 30 µg/mL	
**ANTIOXIDANT**				
PM7 semiempirical method, HAT, SET-PT, SPLET	-	-	4′-OH hydroxyl is the preferred active site, HAT mechanism is energetically the most favored pathway	[78]
DPPH assay, BDPA assay	not given	not given	Kinetic data: 1.4 × 10^2^ k_F_/L mol^−1^ × s^−1^ in MeOH and 1:1 H_2_O/2-propanol (*v*/*v*)	[79]
**ANTICANCER**				
SCC-25 cell line	24, 48 and 72 h incubation in MTT and NRU tests	6.25–200 µM	MTT, IC_50_ > 200 µM NRU, IC_50_ > 200 µM	[80]
C32 cell line			MTT, IC_50_ > 200 µM NRU, IC_50_ > 200 µM	[81]
A375 cell line			MTT, IC_50_ = 100–200 µM NRU, IC_50_ > 200 µM	
inhibitory efficacies against CDK1 through molecular docking	-	-	stable within the binding pocket of the CDK1 protein	[82]
mice with skin tumors	20 weeks	2500 nmol	inhibited the number of tumors per mouse by 16–24% after 15–20 weeks of promotion with TPA	[83]
CCL-220.1 and CCL-222 cell lines (Rhodamine 123 accumulation)	20 min followed by 10 min	1 mg/mL (stock solution)	Fluorescence activity ratio (in CCL-222 MDR1/LRP-expressing cells): at 40 µg/mL—0.83 at 4 µg/mL—1.10 Apoptosis in MDR1 (CCL-222 cells) at 10 µg/mL: early—6.99% apoptosis—4.25% cell death—1.21% Apoptosis in CCL-222 cells early—12.99% apoptosis—4.73% cell death—1.95%	[84]
SW480 and T84 cell lines	48 h incubation	not given	IC_50_ = 100 µM	[85]
**ANTI-MUTAGENIC**				
*Salmonella typhimurium* wit mutagenesis induced by MNU, MNNG, BaP, 2-AA	48 h incubation	not given	% of inhibition: MNU: 11 MNNG: 6 BaP: 1.2 2-AA: 87	[86]
liver microsomes from rats (inhibition of aflatoxin B_1_)	1 h incubation	not given	Microsome-mediated metabolic activation: 11.4% of control; DNA adduct formation of AFB_1_: 7.7% of control.	[87]
**ANTI-NECROPTOSIS**				
MEFs and HT-29 cells	18–20 h incubation	10 µM	IC_50_ = 9.1 µM	[88]
**ENZYME INHIBITION**				
acetylcholinesterase	15 min incubation	50 µL of sample	IC_50_ = 456.48 ± 2.57 µM	[89]
MRP1 and MRP2	0 and 45 min incubation	1, 10, 20, 30, 40 and 50 µM of sample	inhibition at 25 µM: 75% for MRP1 76% for MRP2 2. IC_50_: 13.6 ± 3.9 µM for MRP1 15.0 ± 3.5 µM for MRP2 3. Ki: 5.0 mM for MRP1 8.5 mM for MRP2	[90]
**LIVER DISEASES**				
AML-12 hepatocytes, C57/BL6 mice	18 h incubation with cells mice: 12 weeks	Wester diet (WD) with 0.025%, 0.05% of robinetin	AML-12 cells: ↓ TG accumulation by downregulating lipogenesis-related transcription factors, ↑CD38 expression in OPA-treated cells thought anti-HAT 2. In mice: ↓ mass liver, Improved plasma insulin level and HOMA-IR values ↑ CD38 expression thought anti-HAT ↓ blood glucose levels 3. Computational simulation: dock into the HAT domain pocket of p300, abrogation of its catalytic activity	[91]
liver cells from male Wistar rats	10 min prior to toxic challenge	2.4%	ALT activity: 2.16 IU/g wet cells	[92]

TG—triglyceride; HOMA-IR—homeostatic model assessment of insulin resistance; HAT—histone acetyltransferase; HAT—hydrogentom transfer, SET-PT—single-electron transfer followed by proton transfer; SPLET—sequential proton loss electron transfer; MNU—methylnitrosourea; MNNG—methyl-n-nltro-N-nitrosoguanidine; BaP—benzo-*α*-pyrene; 2-AA—2-aminoanthracene; TPA—12-*O*-tetradecanoylphorbol-13-acetate; FRET—fluorescence resonance energy transfer.

**Table 4:** The selected predicted pharmacokinetic and toxicity properties of robinetin.

Property Name	Prediction Result	Predictive Confidence ^A^
**ABSORPTION**		
human oral bioavailability 20%	non-bioavailable	low
human oral bioavailability 50%	bioavailable	low
human intestinal absorption	absorbed	high
P-glycoprotein inhibitor	non-inhibitor	high
P-glycoprotein substrate	non-substrate	medium
**DISTRIBUTION**		
Blood–Brain Barrier	non-penetrable	high
**METABOLISM**		
CYP 1A2 inhibitor	inhibitor	high
CYP 1A2 substrate	substrate	high
CYP 3A4 inhibitor	non-inhibitor	medium
CYP 3A4 substrate	non-substrate	high
OATP1B1	non-inhibitor	medium
**EXCRETION**		
high-life of drug	<3 h	low
**TOXICITY**		
AMES mutagenesis	safe	medium
eye irritation	toxic	low
carcinogenesis	safe	high
skin sensitization	toxic	medium

^A^ interpretation of predictive confidence available on https://biosig.lab.uq.edu.au/deeppk/theory (accessed on 9 September 2025).

## Data Availability

Data are contained within the article.

## Abbreviations

The following abbreviations are used in this manuscript:

PHF	polyhydroxylated flavonoids
HIV	human immunodeficiency virus
TG	triglyceride
HOMA-IR	homeostatic model assessment of insulin resistance
HAT	histone acetyltransferase
SET-PT	single electron transfer followed by proton transfer
SPLET	sequential proton loss electron transfer
MTT	3-(4,5-dimethylthiazol-2-yl)-2,5-diphenyltetrazolium bromide
NRU	neutral red uptake
DFT	density functional theory
Mpro	main protease
DMSO	dimethyl sulfoxide
HSA	human serum albumin
AD	Alzheimer disease
MeOH	methanol
HPLC	high-performance liquid chromatography
TLC	thin-layer chromatography
Et_2_O	diethyl ether
EtOAc	ethyl acetate
PhA	phosphoric acid
ACN	acetonitrile
NH_4_OAC	ammonium acetate
FA	aormic acid
MDR	multidrug-resistant
MNU	methylnitrosourea
MNNG	methyl-n-nltro-N-nitrosoguanidine
BaP	benzo-*α*-pyrene
2-AA	2-aminoanthracene
TPA	12-*O*-tetradecanoylphorbol-13-acetate
FRET	fluorescence resonance energy transfer
MIC	minimum inhibitory concertation
CDK1	cyclin-dependent kinase 1
BHP	tert-butyl hydroperoxide
CHP	cumene hydroperoxide
MEF	mouse embryonic fibroblasts
WD	western diet
OGTT	oral glucose tolerance test
ADMET	absorption, distribution, metabolism, excretion and toxicity
